# A multimodal framework for early detection and classification of social isolation and loneliness among Chinese older adults

**DOI:** 10.1093/bib/bbaf631.023

**Published:** 2025-12-12

**Authors:** Hao Song, Jueni Lyu, Lu Zhang, Christy M K Cheung

**Affiliations:** Department of Management, Marketing and Information Systems, Hong Kong Baptist University; Department of Management, Marketing and Information Systems, Hong Kong Baptist University; Institute of Systems Medicine and Health Sciences, Hong Kong Baptist University; Department of Computer Science, Hong Kong Baptist University; Institute of Systems Medicine and Health Sciences, Hong Kong Baptist University; Department of Management, Marketing and Information Systems, Hong Kong Baptist University; Institute of Systems Medicine and Health Sciences, Hong Kong Baptist University

## Abstract

**Background:**

Chinese aging population is experiencing growing social isolation and loneliness (SI/L), causing substantial mental concerns. With tremendous efforts devoted to developing interventions, a lack of effective detection hinders the realization of health management for successful aging.

**Methods:**

This study proposes a multimodal SI/L detection and classification framework that integrates multimodal (i.e., linguistic, acoustic, visual, and demographic) data collected from semi-structured interviews to predict the SI/L severity scores of older adults. Specifically, we construct a novel multimodal dataset tailored to the Chinese context. Then, fine-tuned Chinese large language models (i.e., DeepSeek-R1 and BERT-wwm), behavioral signal processing (i.e., OpenFace software), and prompt-based symptom extraction using GPT-5 are employed to generate the 15-dimensional features. These features are then input into a Random Forest regressor to predict SI/L severity scores.

**Findings:**

Preliminary results suggest that multimodal models significantly outperform unimodal and dual-modal approaches in accuracy and robustness, with text-based features playing a dominant role and acoustic/visual cues contributing additional insights. By systematically evaluating single, dual, and multimodal settings, our work highlights the advantages of multimodal integration in improving detection precision.

**Contributions:**

The study proposes a scalable, automated, and linguistically inclusive framework for early SI/L detection among Chinese older adults, addressing current limitations in data quality, population bias, and model interpretability. Our work sheds light on the research of elderly care and the practice in precision and early detection for Chinese older adults’ successful aging.

